# Delays in the diagnosis and management of pulmonary arterial hypertension: a simulated patient cases study across four European countries

**DOI:** 10.1183/23120541.01693-2025

**Published:** 2026-09-01

**Authors:** Fabio Dardi, Brian McCullagh, Filipa Ferreira, Milos Meandzija, Gabriel Stulnig, William Neill, Alejandro Cruz-Utrilla

**Affiliations:** 1 Cardiology Unit, IRCCS Azienda Ospedaliero-Universitaria di Bologna, Bologna, Italy; 2 Mater Misericordiae University Hospital, Dublin, Ireland; 3 Pulmonary Hypertension Unit, Department of Cardiology, Hospital Garcia de Orta, Almada, Portugal; 4 Johnson & Johnson, Belgrade, Serbia; 5 Janssen-Cilag Pharma GmbH, Vienna, Austria; 6 Adelphi Research, Bollington, UK; 7 Pulmonary Hypertension Unit, Department of Cardiology, Hospital Universitario 12 de Octubre, Madrid, Spain; 8 Instituto de Investigación Hospital 12 de Octubre (i+12), Madrid, Spain; 9 ERN-LUNG (European Reference Network on Rare Respiratory Diseases), Frankfurt am Main, Germany; 10 Centro de Investigación Biomédica en Red en Enfermedades Cardiovasculares (CIBER-CV), Madrid, Spain

## Abstract

**Background:**

Pulmonary arterial hypertension (PAH) is progressive and underdiagnosed, contributing to high morbidity and mortality. We assessed physicians’ diagnostic accuracy and management practices using simulated PAH cases across four European countries.

**Methods:**

A cross-sectional online survey (June–August 2024) enrolled 230 consultant cardiologists, pulmonologists and rheumatologists from Germany, Italy, Spain and Portugal. Each completed three simulated PAH cases, representing idiopathic and connective tissue disease (CTD)-associated presentations. Outcomes were correct diagnosis, appropriate referral and adherence to 2022 European Society of Cardiology/European Respiratory Society guideline-recommended management.

**Results:**

Across all 386 cases, the correct diagnosis was made in 49% (190/386). Diagnostic accuracy was higher for idiopathic presentations (59% (80/135)) than CTD-associated presentations (44% (110/251)). Guideline-adherent management occurred in 33% of cases. Among those correctly diagnosing pulmonary hypertension (PH), 32% of physicians failed to follow appropriate referral or testing pathways. Echocardiography was requested in 92% of cases (354/386), but PH was accurately diagnosed in only 52% of those. Physicians in Portugal demonstrated significantly superior diagnostic and management accuracy compared with other countries. Physicians with fewer years of experience (2–10 years) achieved greater diagnostic accuracy than more experienced colleagues.

**Conclusions:**

These findings indicate persistent diagnostic and management variability across four European countries, especially for CTD-associated presentations, and suggest that enhanced echocardiographic training and clearer referral pathways could improve diagnostic accuracy and guideline-adherent care.

## Shareable abstract

**Specialists correctly diagnosed PAH in only half of cases and followed guidelines in one-third. CTD-associated cases were often missed, echo was commonly misread and referral gaps persisted, highlighting urgent needs in training and early recognition.** https://bit.ly/4a9P7WK

## Graphical abstract

**Figure:**
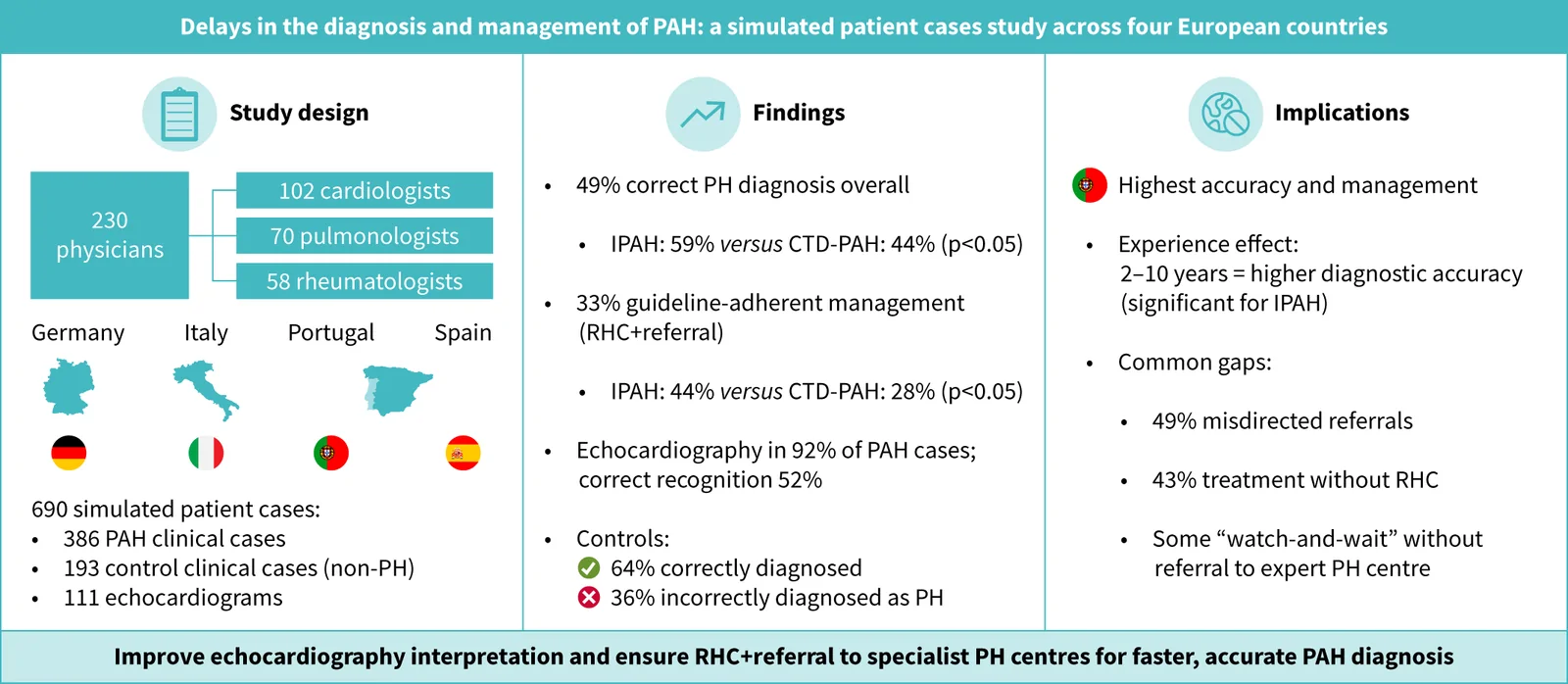
Diagnostic challenges in pulmonary arterial hypertension (PAH). IPAH: idiopathic pulmonary arterial hypertension; CTD: connective tissue disease; RHC: right heart catheterisation; PH: pulmonary hypertension.

## Introduction

Pulmonary hypertension (PH) is a heterogeneous and life-threatening condition characterised by elevated pulmonary arterial pressure, associated with significant morbidity and mortality [1, 2]. Pulmonary arterial hypertension (PAH), a rare subtype of PH, is marked by abnormal pulmonary vascular remodelling leading to right ventricular dysfunction and non-specific symptoms such as exertional dyspnoea, fatigue, chest pain, syncope and palpitations [3, 4]. Although available treatments have improved outcomes, PAH remains underdiagnosed [4, 5].

Median diagnostic delay is estimated at 12–18 months from symptom onset [6, 7]. A key challenge in PAH diagnosis lies in the non-specific symptomatology, often overlapping with other more common cardiopulmonary diseases [5, 6]. Additionally, the increasing epidemiological burden of comorbidities further obscures diagnostic clarity, even when guideline-recommended diagnostic tools are available [8, 9].

Delay or incorrect diagnosis leads to disease progression and worse outcomes [7, 10]. Conversely, early diagnosis improves clinical outcomes [11]. To avoid this prolonged path to an accurate diagnosis, healthcare professionals (HCPs) should undertake a guideline-recommended approach when evaluating patients with PAH-related symptoms [1, 4, 6, 9]. The 2022 European Society of Cardiology (ESC)/European Respiratory Society (ERS) PH guidelines recommend that patients with suspected PAH undergo systematic assessment, including an echocardiogram, and once diagnosed by a confirmatory right heart catheterisation (RHC), referral to a specialist centre is indicated [1]. However, real-world adherence to these recommendations remains inconsistent.

Given these persistent diagnostic challenges, novel methodologies are needed to objectively evaluate clinical decision making. Differently from traditional retrospective approaches, clinical vignette-based simulations provide a validated and standardised approach to examine clinical decision making irrespective of patient-level variability [10, 12, 13]. Using this approach, de Belen *et al*. [10] reported substantial variability in PH evaluation and diagnosis among US cardiologists and pulmonologists, with correct diagnoses in <50% of cases, and limited use of RHC or referral to specialist centres [10], aligning with broader literature on diagnostic variability in conditions with non-specific symptoms [14].

Building on the previous research of de Belen *et al*. [10], the current study employs vignette-based simulations to investigate clinician assessment of PAH and PAH-mimicking conditions across four European countries, extending previous findings beyond the US setting. Specifically, this research explores diagnostic decision-making and referral practices, diagnostic procedures and tests associated with correct diagnosis, as well as differences in practice patterns by specialty (*e.g.* cardiologist *versus* pulmonologist) and by country. Identifying key barriers to guideline-adherent diagnosis, the study aims to generate actionable insights that can inform targeted educational initiatives and potential guideline adaptations to improve diagnostic accuracy and outcomes in PAH.

## Methods

This cross-sectional study was conducted between June and August 2024. An online, quantitative survey utilising simulated patient cases was used to examine the variability and consistency of diagnostic decisions and referral practices in PAH.

### Ethics

The study was conducted in accordance with the ethical standards of Pearl IRB, an independent institutional review board (www.pearlirb.com; Institutional Review Board ID: 2024-0229). All physicians provided informed electronic consent prior to participation and all data were anonymised to ensure confidentiality.

### Study sample (physicians)

Participating physicians were recruited through a structured screening process designed to ensure active clinical involvement and adequate experience in the evaluation of patients with unexplained exertional dyspnoea. Eligibility criteria included board certification or specialist training in cardiology, pulmonology or rheumatology; a minimum duration of clinical practice within the relevant specialty; and a substantial proportion of time devoted to direct patient care. Physicians employed by the pharmaceutical industry were excluded.

To minimise language-related barriers, all materials were translated into the participants’ native languages. The survey was administered online, reflecting a format routinely used in professional medical education and clinical research. A 13-item screening questionnaire was employed to ensure that the sample reflected the broader population of non-PH expert physicians who may encounter patients presenting with PH in clinical practice, while also achieving adequate geographical representation both across participating countries and across different regions within each country.

Participants were required to be fully qualified and actively practising in one of the relevant specialties, with an average of at least 20 h per week dedicated to clinical and patient care activities over the preceding 6 months. Cardiologists and pulmonologists with a specialist focus on pulmonary circulation, pulmonary embolism or right heart failure, as well as rheumatologists working at referral centres for PH or connective tissue disorders, were not eligible to participate.

A target sample size of 240 participating physicians was established based on feasibility considerations. A formal power calculation was not conducted. The final sample comprised 230 physicians.

### Data collection

Data were collected *via* an online survey, designed to take ∼30 min to complete. The survey included demographics, clinical practice and diagnostic decision making. Variables collected included clinical setting, access to RHC, waiting times, collaboration with other specialties, clinical interests and confidence in the 2022 ESC/ERS PH guidelines. Survey design ensured anonymity, and incomplete responses were excluded (no imputation).

### Cases

Participants received nine simulated patient cases, developed by a steering committee of five PH experts (see supplementary table S1 for details). Each participant completed three cases, assigned based on their specialty using a predefined rotation schedule. Cases were presented sequentially, and participants were instructed to manage them as they would in routine clinical practice. The cases were categorised into two types: six clinical cases and three echocardiogram cases (see supplementary table S2 for details).

Each physician completed three simulated patient cases, resulting in 690 total case evaluations. Of these, 386 were PAH cases (instances where PAH was present) and 193 were non-PH control cases. Within the cardiologist subgroup, 37 specialists with echocardiographic expertise completed three echocardiogram cases each, totalling 111 echocardiogram case evaluations (74 PAH echocardiogram cases and 37 non-PH echocardiogram control cases). This distribution ensured adequate representation across different case types while maintaining statistical robustness for the primary analyses. Due to the small number of cases per physician and the exploratory nature of the study, analyses were conducted at the case level without adjustment for intra-physician clustering, similar to previous vignette-based simulation studies in PH [10].

Initially, participants were shown the patient's history and presenting symptoms. Participants were then able to select physical examinations, followed by diagnostic and imaging tests to guide decision making. A detailed overview of each phase of the questionnaire is provided in supplementary table S3. These cases represented either connective tissue disease (CTD)-associated PAH (CTD-PAH) or idiopathic PAH (IPAH). Following case review, physicians were asked to make a diagnosis and describe their next steps (*e.g.* initiate treatment, refer to a specialist, *etc.*).

Echocardiogram cases provided a brief history and video clips of various views. For Cases 7 and 8, physicians gave a diagnosis; for Case 9, they assessed echocardiographic probability of PH and referral for RHC (see supplementary table S4).

Each case was designed with one of three levels of diagnostic complexity: 1) “obvious” PAH cases featured clear symptoms and diagnostic findings that strongly suggested right heart pressure overload; 2) “subtle” cases included mild or non-specific symptoms with only slightly abnormal test results; and 3) control (non-PH) cases mimicked alternative conditions with normal diagnostic findings, acting as comparators. Simulated cases reflected typical PAH presentations and enabled evaluation of decision making across specialties and diagnostic complexity.

### Analysis

The primary objectives of the study were to assess: 1) the proportion of patients correctly diagnosed as having PAH, 2) the extent to which physicians correctly referred diagnosed PAH cases for appropriate follow-up as per the 2022 ESC/ERS PH guidelines recommendations (*e.g.* RHC and referral to specialist centre) and 3) factors contributing to diagnostic accuracy, including the use of specific tests and procedures across clinical and echocardiogram cases.

Descriptive statistics for all numerical variables were summarised using means and standard deviations. p-values were reported to highlight statistical significance of potential trends. Group comparisons were conducted using both univariate and multivariate analyses, with corresponding p-values provided. Comparative analyses focused on differences in diagnostic accuracy and referral patterns between IPAH and CTD-PAH cases. Non-parametric statistical tests, including the Chi-squared test, Cochran's Q-test and the Friedman test, were employed to assess statistical significance (p<0.05) and effect sizes across specialties and countries. Chi-squared tests were additionally used to identify variations in diagnostic and referral practices between countries. All statistical analyses were performed using SPSS Statistics (IBM, Armonk, NY, USA).

## Results

### Sample

A total of 230 physicians (102 cardiologists, 70 pulmonologists and 58 rheumatologists) from Germany, Spain, Italy and Portugal participated in the study (table 1). Together, they completed 690 simulated patient cases. Baseline characteristics of the participating physicians are detailed in table 2.

**TABLE 1:** Study sample

	Germany	Italy	Spain	Portugal	Total
**Cardiologists**	25	28	24	25	102
**Pulmonologists**	17	17	18	18	70
**Rheumatologists**	15	15	15	13	58
**Total**	57	60	57	56	230

Data are presented as n.

**TABLE 2:** Baseline characteristics of physicians

**Sex (at birth)**	
Male	61
Female	38
Prefer not to say	1
**Age, years**	44.7±10.6
**Specialty**	
Cardiology	45
Pulmonologist	30
Rheumatologist	25
**Experience, years**	14.0±7.4
**Time per week providing patient care, h**	40.3±10.8
**Practice type**	
Academic/teaching/university hospital	51
Community/general hospital	22
Specialist centre/referral centre	12
Private hospital	15
**Self-stated confidence in the 2022 ESC/ERS PH guidelines**	
Very confident	56
Somewhat confident	40
Not confident	4
**Work in hospital/centre with capability/facilities to perform RHC testing**	
Yes	69
No	29
Don't know	2

Data are presented as % or mean±sd. ESC: European Society of Cardiology; ERS: European Respiratory Society; PH: pulmonary hypertension; RHC: right heart catheterisation.

### Diagnostic accuracy

Across all cases, PH was correctly diagnosed in 49% (190/386) of PAH cases. Diagnostic accuracy varied by PAH subtype: PH was correctly diagnosed in 59% (80/135) of IPAH cases, while PH was correctly diagnosed in 44% (110/251) of CTD-PAH cases. This difference was statistically significant (p<0.05). No significant differences were found between specialties (table 3).

**TABLE 3:** Correct diagnosis and management of pulmonary arterial hypertension (PAH) cases by country, specialty and case type

	Full sample	Germany	Italy	Spain	Portugal
**Correct diagnosis: all PAH cases (n=386)**	49 (190/386)	43 (41/96)	34 (34/100)	59 (55/94)	63 (60/96)
Cardiologists (n=130)	48 (63/130)	41 (13/32)	33 (12/36)	54 (15/28)	68 (23/34)
Pulmonologists (n=140)	49 (69/140)	44 (15/34)	35 (12/34)	58 (21/36)	58 (21/36)
Rheumatologists (n=116)	50 (58/116)	43 (13/30)	33 (10/30)	63 (19/30)	62 (16/26)
**Correct diagnosis+appropriate management: all PAH cases (n=386)**	33 (129/386)	30 (29/96)	21 (21/100)	30 (28/94)	53 (51/96)*
CTD-PAH cases (n=251)	28 (70/251)	22 (14/63)	20 (13/65)	24 (15/62)	46 (28/61)*
IPAH cases (n=135)	44 (59/135)	45 (15/33)^¶^	23 (8/35)	41 (13/32)	66 (23/35)^#^

Data are presented as % (n/n). PAH: pulmonary arterial hypertension; CTD-PAH: connective tissue disease-associated pulmonary arterial hypertension; IPAH: idiopathic pulmonary arterial hypertension. *: p<0.05 difference *versus* all other countries; ^#^: p<0.05 difference *versus* Italy and Spain; ^¶^: p<0.05 difference *versus* Italy.

When PAH was present but not diagnosed, physicians frequently misattributed symptoms to alternative diagnoses. In CTD-PAH cases, lung diseases (63%) and/or scleroderma (48%) were the most common incorrect diagnoses. In IPAH cases, heart failure/disease or other cardiac conditions were the predominant misdiagnoses (60%).

### Management practices

Appropriate management (defined as both correct diagnosis of PH and implementation of guideline-recommended subsequent actions: referral to a PH expert centre/dedicated PH department and RHC), was observed in 33% (129/386) of PAH cases. Appropriate management was more frequent in IPAH cases (44% (59/135)) compared with CTD-PAH cases (28% (70/251); p<0.05).

Among cases where PH was correctly diagnosed, in 32% (61/190) appropriate follow-up management was not selected. Of these, 49% were incorrectly referred to other specialties instead of PH expert centres (pulmonology (33%), cardiology (23%), rheumatology (23%) and radiology (10%)), 43% were prematurely initiated on treatment without RHC confirmation and 8% were managed with a conservative “watch-and-wait” approach without specialist referral.

No significant differences were observed between specialties: correct diagnosis and management occurred in 34% (44/130) of cardiologists, 36% (50/140) of pulmonologists and 29% (34/116) of rheumatologists.

### Variability across countries

Significant variability in diagnostic and management practices was observed across countries. Physicians in Portugal correctly diagnosed PH more often compared with those from other countries, although differences were not statistically significant (table 3). Physicians in Portugal correctly diagnosed and implemented appropriate management significantly more frequently when compared with other countries for both types of patient cases (table 4).

**TABLE 4:** Characteristics and practice setting of healthcare professionals by country

	Full sample	Germany	Italy	Spain	Portugal
**Grade**					
Consultant	20 (46/230)	67 (38/57)*	0 (0/60)	12 (7/57)^#^	2 (1/56)
Specialist registrar	44 (102/230)	33 (19/57)	25 (15/60)	23 (13/57)	98 (55/56)*
Associate specialist	36 (82/230)	0 (0/57)	75 (45/60)^¶^	65 (37/57)^¶^	0 (0/56)
**Experience, years**					
All	14.1±8.1 (230)	15.4±7.0 (57)^+^	17.2±9.6 (60)^+^	14.4±7.5 (57)^+^	9.1±5.6 (56)
Cardiologists	14.0±7.4 (102)	14.4±6.0 (25)	16.2±8.6 (28)	14.6±6.3 (24)	10.5±7.1 (25)
Pulmonologists	13.6±8.9 (70)	15.1±7.2 (17)	16.8±12.1 (17)	15.1±8.2 (18)	7.7±4.1 (18)
Rheumatologists	14.8±8.5 (58)	17.5±8.0 (15)	19.4±8.6 (15)	13.3±8.6 (15)	8.2±3.4 (13)
**Familiarity with 2022 ESC/ERS PH guidelines**					
Very confident	56 (129/230)	70 (40/57)^+^	60 (36/60)^+^	77 (44/57)^#^	16 (9/56)
Somewhat confident	40 (93/230)	30 (17/57)	40 (24/60)^§^	23 (13/57)	70 (39/56)*
Not confident	3 (8/230)	0 (0/57)	0 (0/60)	0 (0/57)	14 (8/56)*
**Time per week providing patient care, h**	40.3±10.8 (230)	39.8±9.4 (57)	39.1±9.6 (60)	39.8±12.7 (57)	42.6±11.2 (56)
**Practice setting**					
Academic/teaching/university	51 (118/230)	46 (26/57)^+^	48 (29/60)^+^	89 (51/57)*	21 (12/56)
Community/general	22 (51/230)	11 (6/57)	43 (26/60)^ƒ^	5 (3/57)	29 (16/56)^ƒ^
Specialist/referral centre	3 (6/230)	2 (1/57)	5 (3/60)	2 (1/57)	2 (1/56)
Private	14 (33/230)	40 (23/57)*	3 (2/60)	4 (2/57)	11 (6/56)
Outpatient only	13 (30/230)	40 (23/57)*	8 (5/60)^§^	0 (0/57)	4 (2/56)
Mixed inpatient/outpatient	87 (200/230)	60 (34/57)	92 (55/60)^##^	100 (57/57)^¶¶^	96 (54/56)^##^

Data are presented as % (n/n) or mean±sd (n). ESC: European Society of Cardiology; ERS: European Respiratory Society; PH: pulmonary hypertension. *: p<0.05 difference *versus* all other countries; ^#^: p<0.05 difference *versus* Italy and Portugal; ^¶^: p<0.05 difference *versus* Germany and Portugal; ^+^: p<0.05 difference *versus* Portugal; ^§^: p<0.05 difference *versus* Spain; ^ƒ^: p<0.05 difference *versus* Germany and Spain; ^##^: p<0.05 difference *versus* Germany; ^¶¶^: p<0.05 difference *versus* Germany and Italy.

Physicians in Portugal were more frequently specialist registrar, had fewer years of clinical experience and, interestingly, reported lower confidence in the 2022 ESC/ERS PH guidelines. Most physicians in Portugal, Italy and Spain operated in mixed inpatient and outpatient settings, whereas practice settings in Germany were more evenly split between outpatient-only and mixed settings (table 4).

### Factors influencing diagnosis and management

No significant differences were noted between clinical settings (outpatient only *versus* mixed inpatient/outpatient; p=0.39) or physicians’ self-reported confidence in the 2022 ESC/ERS PH guidelines (p=0.51) regarding correct diagnosis and appropriate management. By contrast, experience level appeared to influence diagnostic accuracy and appropriateness of management: physicians with 2–10 years of experience were more likely to correctly diagnose and appropriately manage PAH in both CTD-PAH (35% (40/114) *versus* 27% (21/77) for 11–20 years and 15% (9/60) for >20 years) and IPAH (53% (33/62) *versus* 38% (18/47) for 11–20 years and 31% (8/26) for >20 years). However, this trend reached statistical significance only for IPAH (p<0.05).

### Diagnostic approach

Echocardiography was performed in 92% (354/386) of PAH cases, making it the most frequently requested diagnostic test alongside standard blood tests (90% (346/386)). Despite high utilisation, PH was correctly diagnosed in only 52% (184/354) of cases where echocardiography was performed (table 5).

**TABLE 5:** Diagnostic tests and imaging selected

	Cases in which test requested	PH correctly diagnosed when test requested
**Echocardiography**	92 (354/386)	52 (184/354)
**Blood tests**	90 (346/386)	52 (179/346)
**Chest radiography**	80 (310/386)	51 (157/310)
**Pulmonary function testing**	80 (310/386)	52 (162/310)
**ECG**	73 (280/386)	49 (136/280)
**HRCT/CTPA**	65 (250/386)	56 (139/250)
**Blood tests: advanced**	64 (248/386)	56 (140/248)
**6-min walk test**	49 (188/386)	54 (101/188)
**Cardiopulmonary exercise testing**	35 (134/386)	51 (69/134)
**Ventilation/perfusion scan**	34 (131/386)	67 (88/131)
**Cardiac MRI**	22 (83/386)	59 (49/83)
**Abdominal ultrasound**	19 (75/386)	47 (35/75)
**Capillaroscopy**	19 (75/386)	47 (35/75)
**Coronary CTA**	11 (42/386)	38 (16/32)
**Myocardial SPECT**	4 (15/386)	33 (5/15)
**Pleural ultrasound**	3 (13/386)	23 (3/13)
**Other**	11 (42/386)	67 (28/42)

Data are presented as % (n/n). PH: pulmonary hypertension; HRCT: high-resolution computed tomography; CTPA: computed tomography pulmonary angiography; MRI: magnetic resonance imaging; CTA: computed tomography angiography; SPECT: single-photon emission computed tomography.

### Control cases

A total of 193 control cases were completed. Among patients with symptoms suggestive of PAH but who did not have PAH after evaluation, physicians correctly identified the non-PH primary diagnosis in 64% (124/193) of cases compared with a 49% (190/386) correct diagnosis rate in PAH cases (p<0.05). However, PH was incorrectly diagnosed in 36% (69/193) of control cases. Diagnostic accuracy did not significantly differ between CTD and IPAH control cases (39% (48/123) *versus* 30% (21/70); p=0.09).

In control cases, incorrect suspicion of PH was significantly more frequent when chest computed tomography was requested (71% (49/69) *versus* 55% (68/124); p=0.03) and when capillaroscopy was requested (30% (21/69) *versus* 15% (18/124); p=0.01). An echocardiogram was requested in 95% of control cases.

Interestingly, among physicians who incorrectly suspected PH in control cases, 22% reported that they would request additional “other” tests to support the diagnosis; of these, 13 out of 15 spontaneously indicated cardiac catheterisation. In contrast, among physicians who suspected an alternative diagnosis, only 8% reported requesting additional tests and only one out of 10 indicated catheterisation.

### Impact of case sequence (rotation bias)

The potential influence of case order on diagnostic accuracy was assessed and found to be non-significant (α=0.05). When a control case was presented first, 50% of subsequent PAH cases were correctly diagnosed compared with 46% when an obvious PAH case was first and 37% when a subtle PAH case was first (p=0.27). When a control case was presented first, 42% were incorrectly diagnosed as PH compared with 32% when an obvious PAH case was first, 32% when a subtle PAH case was first and 33% when two PAH cases were presented first (p=0.58). These findings suggest no systematic bias related to case sequence.

### Echocardiogram cases

Among the small subset of specialist cardiologists (n=37) trained in echocardiography who completed the echocardiogram-based cases, approximately two-thirds correctly identified PH on the provided echocardiographic images/videos. In Case 7, PH was correctly diagnosed 65% (23/37) of the time, with cardiologists from Spain demonstrating the highest recognition rates (90% (9/10)). Notably, those with 2–10 years of experience were more likely to diagnose PH (77% (10/13)) compared with those with more years of experience (table 6).

**TABLE 6:** Case 7 echocardiogram case diagnosis

	PH diagnosis	Other diagnosis
**Full sample**	65 (23/37)	35 (14/37)
**Country**		
Germany	67 (6/9)	33 (3/9)
Italy	40 (4/10)	60 (6/10)
Spain	90 (9/10)	10 (1/10)
Portugal	63 (5/8)	38 (3/8)
**Experience, years**		
>20	20 (1/5)	80 (4/5)
11–20	68 (13/19)	32 (6/19)
2–10	77 (10/13)	23 (3/13)

Data are presented as % (n/n). PH: pulmonary hypertension.

In Case 9, 62% (23/37) of cardiologists categorised the patient as having intermediate or high echocardiographic probability of PH and would refer for an RHC.

For both Cases 7 and 9, tricuspid regurgitation continuous wave Doppler was the most selected additional echocardiographic parameter. Among those who selected this parameter, 89% (17/19) in Case 7 and 88% (22/25) in Case 9 subsequently provided the correct diagnosis and/or appropriate management. Table 7 summarises the additional echocardiographic views selected.

**TABLE 7:** Echocardiogram views selected by all physicians and by the physicians making the appropriate diagnosis

	Case 7		Case 9	
	Cases in which test requested	PH correctly diagnosed when test requested	Cases in which test requested	PH correctly diagnosed/managed when test requested
**Tricuspid regurgitation continuous wave Doppler**	51 (19/37)	89 (17/19)	68 (25/37)	88 (22/25)
**TAPSE**	49 (18/37)	83 (15/18)	54 (20/37)	85 (17/20)
**Mitral pulse wave Doppler**	27 (10/37)	80 (8/10)	38 (14/37)	86 (12/14)
**Left ventricle tissue Doppler**	24 (9/37)	78 (7/9)	27 (10/37)	90 (9/10)
**Pulmonary artery diameter**	22 (8/37)	75 (6/8)	30 (11/37)	82 (9/11)
**Right ventricular outflow tract pulse wave Doppler**	16 (6/37)	100 (6/6)	27 (10/37)	100 (10/10)
**Interatrial septum/pulmonary venous return**	16 (6/37)	50 (3/6)	15 (5/37)	80 (4/5)

Data are presented as % (n/n). TAPSE: tricuspid annular plane systolic excursion.

In Case 8 (the echocardiogram control case, in which PH was not present), PH was incorrectly suspected in 11% (4/37) of cases, while the majority of cardiologists (89% (33/37)) correctly identified the absence of PH.

## Discussion

This study highlights persistent diagnostic delays and notable variability in clinical practice when evaluating patients with PAH. Using simulated patient cases across four European countries, we found inconsistencies in clinical decision making and adherence to guideline-recommended management, closely mirroring a similar US-based study [10].

PAH was correctly diagnosed in only 49% of cases, equal to the US rate [10]. Non-specific presentations and symptom overlap with common cardiopulmonary diseases often obscure the diagnosis, leading to delays and misdiagnoses [5, 8, 10]. Symptoms were frequently misattributed to heart failure or other complications of CTD, further impacting outcomes [11].

Diagnostic accuracy differed by PAH subtype: IPAH was more often correctly diagnosed (59%) than CTD-PAH (44%). In CTD-PAH, physicians often attributed symptoms to rheumatological conditions or CTD-related lung disease, overlooking PAH as a complication. Physicians with 2–10 years of experience demonstrated greater diagnostic accuracy. This observation may reflect, at least in part, more recent training and familiarity with contemporary diagnostic approaches, consistent with prior evidence suggesting an association between physician experience and clinical outcomes [15]. Increased awareness of PAH in recent years, during the training period of younger physicians, may also have contributed.

These findings suggest a need for integrated education. Initiatives should enhance CTD-PAH recognition in rheumatology and primary care curricula, with clear referral pathways for at-risk patients. Professional societies should support continuing education for experienced clinicians, ensuring rare diseases like PAH remain in focus. Programmes should emphasise practical application of diagnostic approaches and echocardiographic markers.

Notably, appropriate guideline-adherent management, defined as correct diagnosis of PH and follow-up actions such as RHC and referral to a specialist PH centre, occurred in only 33% of cases, similar to previous research (41% in the US study) [10]. Almost one-third of cases with a correct PAH diagnosis did not receive appropriate follow-up. This gap reflects lapses in applied knowledge, with physicians at times initiating treatment without RHC, adopting a “watch-and-wait” approach or misdirecting referrals to non-specialist departments.

Systemic factors may also contribute to these shortfalls. About half of participating physicians reported working in hospitals capable of performing RHC. However, RHC capability must be linked with comprehensive, guideline-compliant diagnostic workups at specialist PH centres, which integrate both diagnostic and management expertise. Appropriate referral has downstream implications for diagnostic quality and patient outcomes.

Diagnostic test selection strongly influenced performance. Echocardiography was requested in most cases (92%), yet PH was correctly diagnosed in half of these patients [10]. This points to challenges in its interpretation. Key parameters recommended by the 2022 ESC/ERS PH guidelines, such as tricuspid regurgitation continuous wave Doppler, TAPSE (tricuspid annular plane systolic excursion) and right ventricular outflow tract Doppler, were frequently overlooked [1, 8, 9], reducing diagnostic accuracy. These gaps highlight the need for practical training focused on recognising and applying echocardiographic markers that reliably predict PH.

Country-level variation in diagnostic accuracy and management was observed, with physicians in Portugal demonstrating higher diagnostic accuracy and greater adherence to guideline-recommended management. These findings emerged despite lower self-reported confidence in the 2022 ESC/ERS PH guidelines. Potential contributing factors may include the concomitance of a higher representation of physicians with fewer years of experience, as discussed earlier, or in specialist training, who may be more exposed to structured educational activities, as well as a predominantly inpatient working context, which may facilitate access to comprehensive diagnostic pathways. Although speculative and not disentangled by the present study design, these observations are consistent with previous literature highlighting the role of institutional context and training environment in PH care [16].

Finally, the misclassification of 36% of control cases as PH represents a clinically relevant finding, as over-suspicion may led to unnecessary investigations, referrals and invasive procedures. In the present study, incorrect PH diagnoses were more frequently associated with requests for advanced diagnostic tests, despite first-line examinations, such as echocardiography, being not suggestive of PH. Consistent with previous observations, physicians were more accurate in identifying alternative, non-PH diagnoses than in confirming PH [10]. Although the overall rate of incorrect PH diagnosis was lower than previously reported [10], our results suggest that once PH enters the differential diagnosis, the threshold for pursuing PH-directed invasive investigations may decrease, potentially at the expense of integrating non-invasive test results. The absence of a significant case-sequence effect suggests that these findings are unlikely to be driven by artefacts of vignette presentation.

Several limitations should be acknowledged. Clinical vignette-based simulations cannot fully replicate real-world clinical care or capture longitudinal symptom evolution, iterative assessments and qualitative patient–physician interactions. In addition, system-level factors, such as resource availability, healthcare infrastructure and reimbursement policies, were not explicitly addressed. Nevertheless, the standardised nature of the simulated cases represents a methodological strength, as it enables controlled comparisons of diagnostic approaches while minimising confounding related to case-mix variability.

Future research should build on these results by exploring educational interventions aimed at improving both clinical and echocardiographic interpretation skills, including the potential role of artificial intelligence-supported tools, as well as system-level factors, such as guideline implementation, institutional pathways and healthcare system characteristics.

In conclusion, our findings emphasise persistent diagnostic challenges and variability in PAH management within Europe, echoing the US findings. High-quality echocardiographic assessment remains critical for early diagnosis, reinforcing the need for targeted training. Structured, practical education and clearer implementation of multidisciplinary pathways are key to improving diagnostic accuracy and treatment outcomes. The cost-effectiveness of such strategies may be maximised by prioritising educational outreach to those most likely to encounter PAH, including physicians managing patients in the major risk groups outlined in the 2022 ESC/ERS PH guidelines (systemic sclerosis, other CTDs, HIV infection, congenital heart disease, portal hypertension or a family history of PAH).

## Data Availability

The datasets generated and analysed during the current study are not publicly available, but are available from the corresponding author on reasonable request. Aggregated anonymised summary data (*e.g.* counts, percentages and group-level results) may be provided subject to approval by the steering committee and Johnson & Johnson.

## Supplementary material

**Please note:** supplementary material is not edited by the Editorial Office, and is uploaded as it has been supplied by the author. Supplementary material 01693-2025.SUPPLEMENT
