# Supplementary material for: Delays in the diagnosis and management of pulmonary arterial hypertension: a simulated patient cases study across four European countries
Source: ERJ Open Res. 2026 Sep 1;12(5):01693-2025. doi: 10.1183/23120541.01693-2025 (PMC13531297; doi:10.1183/23120541.01693-2025)

Supplement

Table S1: Steering committee members – overview

Name	Overview
Dr Fabio Dardi	Specialty: Cardiologist (Consultant)<br>Country: Italy<br>Involvement with PH: Dr. Dardi is a cardiologist at the IRCCS Sant'Orsola University Hospital of Bologna and has completed a PhD program in 2021.<br>Alongside his current role as a cardiologist, Dr. Dardi coordinates a research group at the Bologna centre focusing on PH.
Dr Filipa Ferreira	Specialty: Cardiologist<br>Country: Portugal<br>Involvement with PH: Dr. Ferreira is the coordinator of the PH unit since 2019 at Hospital Garcia de Orta - a national referral Centre for PH.<br>Previously Dr. Ferreira has performed a fellowship on congenital heart disease with Professor Michael Gatzoulis and a Master's degree focusing on PH. PH is an area of particular interest for Dr. Ferreira, being involved with several publications, lectures, and educational initiatives.
Dr Alejandro Cruz Utrilla	Specialty: Cardiologist<br>Country: Spain<br>Involvement with PH: Dr. Utrilla is a physician and researcher who has focused exclusively on PH since 2019. His work has looked at the genetics of PAH, and the correlation between the clinical aspects and the genetic background of the disease. Dr. Utrilla is an active member of the Spanish PH group and collaborates with members associations and patients to spread the current knowledge and innovations of this rare disease.
Dr Brian McCullagh	Specialty: Pulmonologist<br>Country: Ireland<br>Involvement with PH: Dr. McCullagh is a member of the National PH unit in the Mater Misericordiae University Hospital which is the only specialised unit for PH in Ireland. He completed his MD thesis on growth factors and chemokines in Pulmonary Hypertension and along with managing PH patients is actively involved in collaborative clinical research and patient advocacy.

Table S2: Simulate patient cases - description

#	Case types	Case variant	Case description
**1**	Connective tissue disease (CTD) cases	Systemic sclerosis (SSc) + pulmonary arterial hypertension with symptoms (obvious)	• Symptomatic for exertional dyspnoea and/or findings on diagnostic testing (e.g., echocardiography, laboratory, or imaging) that clearly indicate right heart overload with values that deviate significantly from normal limits.
**2**		Systemic sclerosis (SSc) + pulmonary arterial hypertension without symptoms (subtle)	• Either asymptomatic or findings on diagnostic testing (e.g., echocardiography, laboratory, or imaging) that indicate right heart overload but deviate only slightly from normal limits.
**3**		Systemic sclerosis (SSc) non-pulmonary hypertension with symptoms (control)	• Symptoms but findings on diagnostic testing (e.g., echocardiography, laboratory, or imaging) are within normal limits (i.e., no evidence of right heart overload).
**4**	Idiopathic pulmonary arterial hypertension (IPAH) cases	Idiopathic pulmonary arterial hypertension with clear clinical picture (no comorbidities) (obvious)	• Symptoms such as dyspnoea (without comorbidities that justify symptoms) and findings on diagnostic testing (e.g., echocardiography, laboratory, or imaging) that clearly indicate right heart overload with values that deviate significantly from normal limits.
**5**		Idiopathic pulmonary arterial hypertension with unclear clinical picture (with comorbidities) (subtle)	• Dyspnoea in the context of comorbidities that could explain symptoms and/or findings on diagnostic testing (e.g., echocardiography, laboratory, or imaging) that indicate right heart overload but deviate only slightly from normal limits.

**6**		Non pulmonary hypertension displaying symptoms (with comorbidities)<br>(control)	• Symptoms but findings on diagnostic testing (e.g., echocardiography, laboratory, or imaging) are within normal limits (i.e., no evidence of right heart overload).
**7**	Echocardiogram (ECHO) cases	Systemic sclerosis (SSc) + pulmonary arterial hypertension without symptoms<br>(subtle)	• Echocardiographic findings that clearly indicate right heart overload with values that deviate significantly from normal limits.
**8**		Non-pulmonary hypertension displaying symptoms (due to other cardio-pulmonary conditions) with a low probability of PH<br>(control)	• Echocardiographic findings that indicate right heart overload but deviate only slightly from normal limits.
9		Idiopathic pulmonary arterial hypertension with clear clinical picture (no comorbidities)<br>(obvious)	• Echocardiographic findings are within normal limits (i.e., no evidence of right heart overload).

Table S3: Clinical cases (1–6) overview at each stage

Round	Options	Notes
Basic patient information	Age<br>Gender<br>Background<br>Symptoms<br>General inspection overview<br>Current medication	• Provided to all respondents.
Patient history	Diabetes Mellitus<br>Obesity<br>Hypertension<br>Hypotension<br>Venous thromboembolism (VTE)<br>Heart Disease<br>Atrial fibrillation<br>Asthma<br>Lung Disease<br>Allergies<br>Systemic sclerosis<br>Gastrointestinal disease<br>Metabolic disease<br>Endocrine disorder(s)<br>Mood disorder(s)<br>Patient smoking status<br>Activity level / exercise route<br>Dietary information<br>Occupation / current exercise routine<br>Family medical history	• Respondents could select between 5 and 7 of these pieces of information they would like to learn more about the patient's medical history.<br>• For each selected, information will be provided to state if they have any history with these factors.<br>• Results were then shown on the next page.<br>• All respondents viewed this question.
Physical examinations	Blood pressure<br>Temperature<br>Heart rate<br>Oxygen saturation<br>Breathing rate/ respiratory rate<br>Respiratory exam<br>Cardiovascular examination<br>Gastrointestinal examination/ digestive examination<br>Genito-urinary exam<br>Neural Exam<br>Lymph nodes<br>Ocular/ eye exam<br>Auditory / ear exam	• Respondents could select as many factors as they wanted.<br>• Results were then shown on the next page.<br>• All respondents view this question.
Round 1: Laboratory tests, imaging studies, and diagnostic procedures	Blood tests:<br>• Complete blood count (CBC)<br>• Comprehensive metabolic panel (CMP) - renal and liver profile<br>• Thyroid function (TSH, T4 or T3)<br>Electrocardiogram (ECG)<br>Echocardiogram<br>Chest radiography (X-ray)<br>Abdominal ultrasound	• Respondents could select up-to 5 tests they wish to conduct.<br>• Results were then shown on the next page.<br>• Following review of the results of these tests, respondents then had the option to either request further tests or move directly to making a diagnosis.

	Pulmonary Function Test (PFT)<br>Cardiopulmonary Exercise Test (CPET)<br>6-min walk test (6MWT)	• All respondents viewed this question.
Round 2: Laboratory tests, imaging studies, and diagnostic procedures	All tests **not selected at round 1**, plus:<br>“Advanced” blood tests:<br>• Troponin (cTn),<br>• Arterial blood gas (ABG)<br>• Nucleic Acid Test (NAT) – HIV<br>• Natriuretic Peptide Tests (BNP, NT-proBNP)<br>• Erythrocyte sedimentation rate (ESR) and C-reactive protein (CRP)<br>High Resolution Computed Tomography (HRCT)/ Computed Tomography Pulmonary Angiogram (CTPA)<br>Coronary CT scan<br>Myocardial perfusion single proton emission computed tomography (SPECT)<br>Cardiac magnetic resonance imaging (MRI)<br>Pleural ultrasound<br>Lung ventilation/perfusion (V/Q) scan<br>Capillaroscopy	• Respondents could select up-to 5 tests they wish to conduct.<br>• Results were then shown on the next page.<br>• Following review of the results of these tests, respondents then had the option to either request further tests or move directly to making a diagnosis.<br>• Only those respondents who requested more tests at Round 1.
Round 2: Laboratory tests, imaging studies, and diagnostic procedures	All tests not selected at round 1 or 2.	• Respondents could select up-to 1 further test they wish to conduct.<br>• Results were then shown on the next page.<br>• Following review of the results of these tests, respondents move directly to making a diagnosis.<br>• Only those respondents who requested more tests at Round 2.

Table S4: Echocardiogram cases (7–9) overview at each stage

Round	Options	Notes
Basic patient information	Age<br>Gender<br>High-level overview of symptoms<br>Co-morbidities<br>Current medications	• Provided to all respondents.
Basic echocardiogram results	Parasternal long axis view (PLAX) – Left Ventricle<br>Parasternal short-axis (PSAX) - Aorta<br>Parasternal short-axis (PSAX) – Papillary Muscles<br>4 chamber apical view<br>4 chamber apical view – Colour Doppler<br>Subcostal view	• Descriptions and videos of each.<br>• Provided to all respondents.<br>• Following review of these echocardiogram views, respondents then had the option to either request further views or move directly to making a diagnosis.
Echocardiogram additional views (round 1)	Tricuspid Annular Plane Systolic Excursion (TAPSE)<br>Mitral Pulse Wave (PW) Doppler<br>Left Ventricle Tissue Doppler (TDI)<br>Tricuspid Regurgitation (TR)<br>Continuous Wave (CW) Doppler<br>Right Ventricular Outflow Tract (RVOT) Pulse Wave (PW) Doppler<br>Pulmonary Artery Diameter<br>Interatrial Septum and Pulmonary Venous Return	• Respondents could select up-to 3 views they wish to review.<br>• Views were then shown on the next page.<br>• Following review of the results of these views, respondents then had the option to either request further views or move directly to making a diagnosis.<br>• Only those respondents who requested more tests at "Basic echocardiogram results".
Echocardiogram additional views (round 2)	All views not selected at echocardiogram additional views (round 1)	• Respondents could select up-to 4 views they wish to review.<br>• Views were then shown on the next page.<br>• Following review of the results of these views, respondents then moved directly to making a diagnosis.<br>• Only those respondents who requested more tests at "Basic echocardiogram results".

S1. Questionnaire flow (clinical cases)

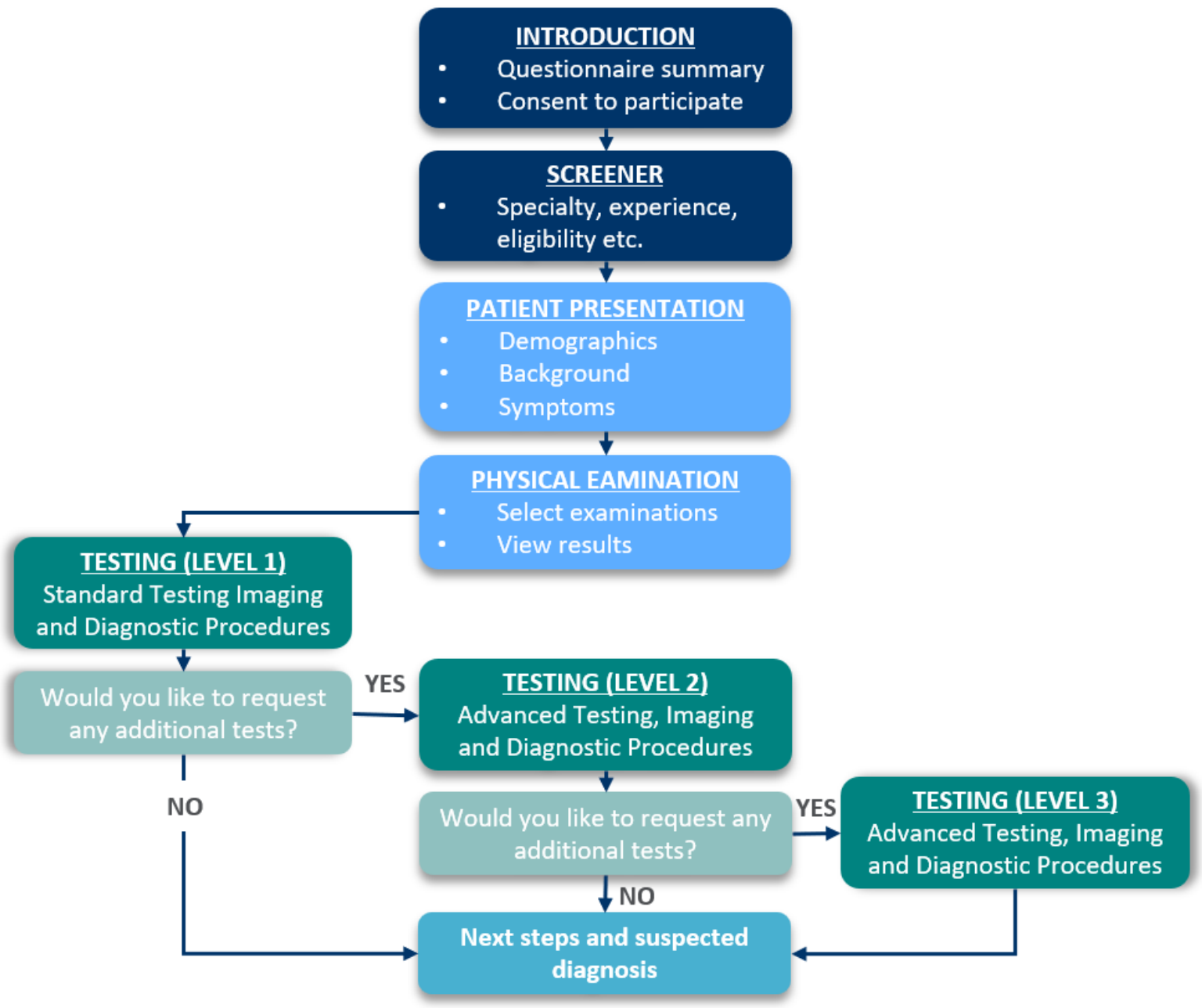

S2. Questionnaire flow (echocardiogram cases)

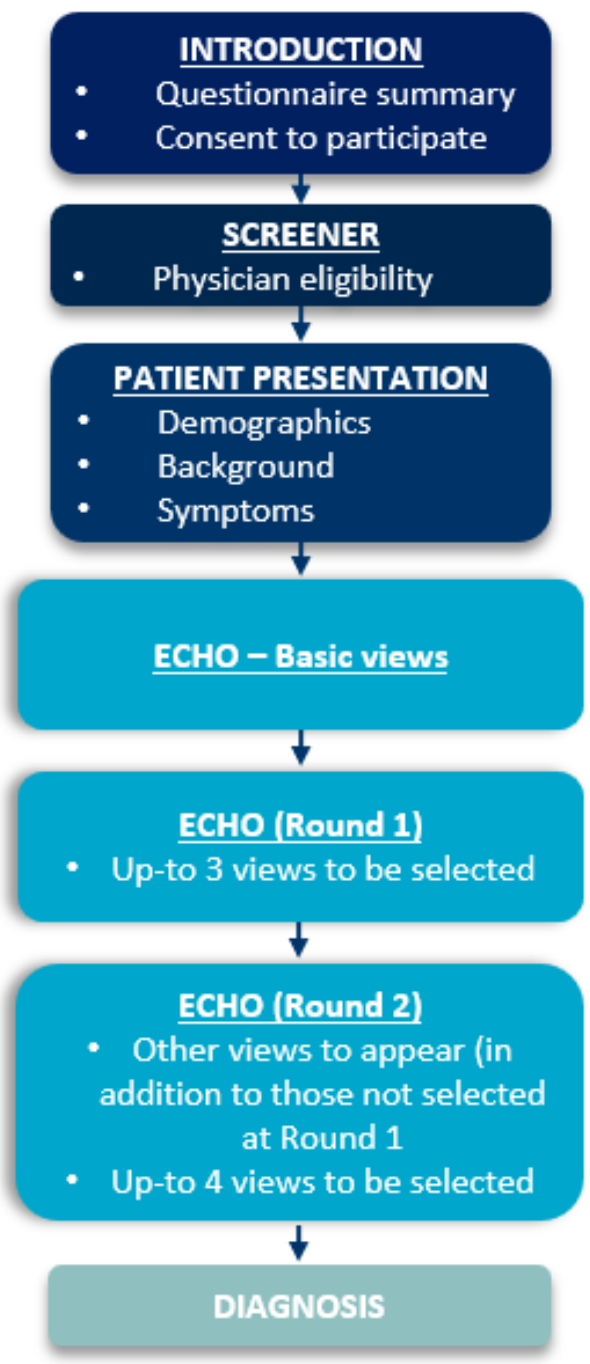

Supplement: Supplementary file 1 [file 01693-2025.SUPPLEMENT.pdf]
